# Budesonide inhalation suspension versus methylprednisolone for treatment of moderate bronchial asthma attacks

**DOI:** 10.1186/s40413-015-0065-0

**Published:** 2015-05-05

**Authors:** Noriyuki Yanagida, Morimitsu Tomikawa, Akinori Shukuya, Masamichi Iguchi, Motohiro Ebisawa

**Affiliations:** Department of Pediatrics, Sagamihara National Hospital, Kanagawa, Japan; Clinical Research Center for Allergy and Rheumatology, Sagamihara National Hospital, Kanagawa, Japan

**Keywords:** Asthma, Methylprednisolone, Budesonide, Inhalation, Cortisol, Procaterol

## Abstract

**Background:**

Owing to their side effects, administration of steroids for bronchial asthma attacks should be minimized. We investigated whether budesonide inhalation suspension (BIS) could replace intravenous steroid administration for the treatment of moderate bronchial asthma attacks.

**Subjects and Methods:**

The subjects were children aged 5 years and younger hospitalized for moderate bronchial asthma attacks. Patients were randomly assigned to one of two groups: 20 patients received methylprednisolone (mPSL) and 20 were treated with BIS. The mPSL group began treatment with inhalation of procaterol hydrochloride (0.3 mL) and disodium cromoglycate (2 mL) three times a day and systemic administration of mPSL (1 mg/kg) three times a day. The BIS group began treatment with inhalation of procaterol hydrochloride (0.3 mL) and BIS (0.5 mg) three times a day. The frequency of inhalations and steroid administration was adjusted according to the severity of symptoms. The cortisol level at discharge was measured.

**Results:**

There were no significant differences between the two groups in terms of the severity of attacks and duration of management, or in terms of therapeutic efficacy, duration of wheezing, or period of hospitalization. The frequency of inhalations on days 3 to 6 of hospitalization was lower in the BIS group than in the mPSL group, and the cortisol level at discharge was significantly higher in the BIS group (13.9 ± 6.1 μg/dL) than in the mPSL group (8.0 ± 2.1 μg/dL) (p = 0.008).

**Conclusion:**

In patients with recurrent wheezing or bronchial asthma of <5 years, the efficacy of BIS is equivalent or better than mPSL for moderate bronchial asthma attacks, and in contrast to steroid treatment, BIS treatment do not suppress adrenocortical function.

## Background

Administration of steroids for bronchial asthma attacks should be minimized to prevent adverse effects. Budesonide inhalation suspension (BIS) is used for the long-term management of bronchial asthma in children aged from 6 months to 5 years [1]. There have been a few reports on the use of BIS in patients with acute asthma attacks, but a consensus is yet to be reached on its role in the treatment of these patients. The recommended dose of BIS has previously been reported to be 0.8 to 2 mg per administration, which is higher than the maximum dose of 0.5 mg normally used in Japan [2]. If BIS can be generally administered at home for treatment of asthma attacks, the medical costs associated with hospitalization for bronchial asthma attacks would likely be reduced.

The aim of the present study was to investigate whether BIS at a dose of 0.5 mg could be used for the treatment of moderate bronchial asthma attacks rather than steroid injection.

## Methods

### Definition of bronchial asthma

In infantile asthma, diagnosis is based mainly on clinical data. Furthermore, some small children have recurrent episodes of acute wheezing during respiratory infections only at this age and do not actually have persistent asthma later in their life. In the Japanese Pediatric Guideline for the Treatment and Management of Bronchial Asthma 2008 (JPGL2008), for early intervention, a diagnosis of infantile asthma can be made if there are 3 or more episodes of marked expiratory wheezing, regardless of the presence of respiratory tract infection. These criteria may include wheezing caused by viral infection; therefore, we used this definition. Moderate bronchial asthma attacks were also defined by the JPGL2008 as the presence of apparent wheezing, retractive breathing, prolonged expiration orthopnea, or increased respiratory rate.

### Subjects

#### Inclusion criteria

All subjects of this study were patients aged 5 years and younger admitted to the Department of Pediatrics at Hospital between October 2008 and March 2010 because of moderate bronchial asthma attacks.

#### Exclusion criteria

Patients who required oxygen on admission and patients with respiratory syncytial virus infection were excluded. A rapid respiratory syncytial virus antigen detection kit was used for diagnosis of respiratory syncytial virus infection in all patients in admission. Oxygen was administered if the percutaneous oxygen saturation (SpO_2_) was 92% or lower. Patients admitted on weekends, national holidays, or at night were also excluded. We did not consider intravenous steroid treatment prior to admission as an exclusion criterion.

#### Outcome of the study

The primary outcome of this study was the Modified Pulmonary Index Score (MPIS) during hospitalization, and the secondary outcome was cortisol level at discharge.

#### Methods of treatment

After written consent had been obtained, the envelope method was used to randomly assign 20 patients to the methylprednisolone (mPSL) group and 20 to the BIS group. The mPSL group began the initial treatment with inhalation of a mixture of 0.01% procaterol hydrochloride (0.3 mL) and 1% disodium cromoglycate (2 mL) three times a day and intravenous administration of mPSL (1 mg/kg) three times a day. The BIS group began the initial treatment with inhalation of a mixture of 0.01% procaterol hydrochloride (0.3 mL) and BIS (0.5 mg, 2 ml) three times/day. Maintenance fluid infusions were given to all patients in both groups. Disodium cromoglycate is used as maintenance treatment for children with moderate asthma in Japan [3]. Procaterol hydrochloride is a bronchodilator that is widely used in Japan [4]. The JPGL2008 recommends a dose of 0.1 to 0.3 mL for infants and preschool children, and 0.2 to 0.4 mL for school-age children [1]. In this study, 0.3 mL of procaterol hydrochloride was administered regardless of age. The frequency of using an inhaled bronchodilator, BIS or mPSL was increased or reduced as appropriate in accordance with symptoms such as wheezing and dyspnea independently. The jet inhalation device used was a Micro Mist Nebulizer® (Hudson RCI/Teleflex, Research Triangle Park, NC, United States). In principle, inhalation was performed by a nurse; if performed by a family member, it was monitored by a nurse to ensure that appropriate procedures were followed. Parameters such as pulse rate, SpO_2_, respiratory rate, wheezing, use of respiratory musculature, and retracted breathing were evaluated on a daily basis during hospitalization. The Modified Pulmonary Index Score (MPIS) was used to assess the severity of attacks that required hospitalization and the patient’s respiratory status during hospitalization [5]. Atopic bronchial asthma was defined as positive immunoglobulin E (IgE) antibodies for *Dermatophagoides pteronyssinus* (≥0.35 UA/mL by the ImmunoCAP method, Thermo Fisher Scientific Inc., Uppsala, Sweden). Blood tests and eosinophil cationic protein (ECP) and cortisol level measurements were performed on admission and at discharge. Cortisol levels were measured in blood sampled before noon on the day of admission and 10 a.m. on the day of discharge. Cortisol levels at discharge were measured in blood sampled at least 2 days after final steroid administration. No blood samples were taken if a patient was discharged on a weekend or national holiday. We compared the duration of hospitalization (in days), the frequency of using an inhaled bronchodilator, and plasma cortisol levels at discharge.

#### Statistical method

The results are shown as means ± standard deviations. The *t*-test and the Mann–Whitney *U* test were used to test for significance, and the Fisher exact test was used to test proportions. In all cases, *p* <0.05 was regarded as significant. Statistical Package for Social Science (SPSS) 20.0 (IBM, Armonk, NY, United States) was used for statistical analysis. Mite-specific IgE was calculated by the geometric mean.

#### Ethical considerations

After collecting data on all patients, the study was registered as a clinical trial. Written informed consent was obtained from the legal guardians of all patients, and the study protocol was approved by the Ethics Committee of the institute.

## Results

1. *Patient characteristics*

Of the 70 subjects (46 male and 24 female), 13 who required oxygen on admission and four with respiratory syncytial virus infection were excluded. Oxygen was administered if percutaneous oxygen saturation (SpO_2_) was 92% or lower. Thirteen patients admitted on weekends, national holidays, or at night were also excluded; thus, 40 patients were finally included as subjects.

Table 1 shows a comparison of patient characteristics prior to admission. There were no significant differences between the two groups in terms of age, male-to-female ratio, type of bronchial asthma (atopic or other), severity based on bronchial asthma treatment on admission, passive smoking, or pet-keeping. Fourteen subjects (70%) were positive for dust mite-specific IgE in both the mPSL and BIS groups, and most subjects in both groups suffered from atopic bronchial asthma.

**Table 1:** **Patient characteristics**

	**mPSL group**	**BIS group**	***p***
	**(n = 20)**	**(n = 20)**	
Age (years)	3.1 ± 1.4	3.1 ± 1.3	n.s.
Sex	Male: 14	Male: 13	n.s.
	Female: 6	Female: 7	
Cases of atopic asthma	14	14	n.s.
Mite-specific IgE (UA/mL)	6.3 ± 21.0	8.9 ± 22.7	n.s.
Regular treatment for asthma prior to admission	None: 5	None: 6	n.s.
	Montelkast or Pranlukast: 15	Montelkast or Pranlukast: 14	
	Inhaled corticosteroids: 10	Inhaled corticosteroids: 6	
Passive smoking	5	6	n.s.
Pet-keeping	4	2	n.s.

JPGL2008, Japanese pediatric guideline for the treatment and management of asthma 2008; mPSL, methylprednisolone; BIS, budesonide inhalation suspension; ECP, eosinophil cationic protein; n.s., not significant.

Table 2 shows a comparison of patients’ vital signs and hematological data on admission. There were no significant differences between the two groups in terms of physical signs such as pulse rate, SpO_2_, and temperature, or in terms of blood test results such as white blood cell count or C-reactive protein, cortisol, or ECP levels. Asthma severity on admission was compared according to the MPIS. There were no significant differences between the two groups in terms of the total score or individual scores for respiratory rate, pulse rate, SpO_2_, wheezing, or use of respiratory musculature. Moreover, there were no significant differences between the two groups in terms of patient characteristics and severity of attacks. Ten patients in the mPSL group and eight in the BIS group had already received intravenous steroid treatment prior to admission, but the difference was not significant. There was no use of oral or other intravenous steroids. None of the patients received multiple treatments with hydrocortisone sodium succinate, and the maximum dose was 7 mg/kg.

**Table 2:** **Comparison of physical and laboratory findings on admission**

	**mPSL group**	**BIS group**	***P***
	**(n = 20)**	**(n = 20)**	
Heart rate (beats/min)	133.3 ± 14.6	135.0 ± 18.1	n.s.
SpO_2_ (%)	96.7 ± 0.8	95.9 ± 1.3	n.s.
Temperature (°C)	37.2 ± 0.8	37.2 ± 0.7	n.s.
White blood cells (/mL)	10487 ± 3508	11604 ± 3941	n.s.
CRP (mg/dL)	0.8 ± 0.9	0.7 ± 0.6	n.s.
Cortisol (mg/dL)	85.0 ± 101.6	56.8 ± 76.0	n.s.
ECP (mg/L)	39.7 ± 35.8	25.7 ± 19.8	n.s.

mPSL, methylprednisolone; BIS, budesonide inhalation suspension; CRP, C-reactive protein; SpO_2_, percutaneous oxygen saturation; ECP, eosinophil cationic protein; n.s., not significant.

2) *Treatment details*

Table 3 shows a comparison of treatment details and test data during hospitalization. There were no significant differences between the two groups in terms of the duration of wheezing, duration of BIS or mPSL, or hospitalization time. The mean total dose of mPSL administered in the mPSL group was 158 ± 96 mg, and the mean total dose of BIS administered in the BIS group was 5.0 ± 1.6 mg, although these values cannot be compared because of the different routes of administration and formulations used.

**Table 3:** **Comparison of treatment details**

	**mPSL group**	**BIS group**	***p***
	**(n = 20)**	**(n = 20)**	
Duration of wheezing (days)	4.5 ± 1.3	4.0 ± 1.6	n.s.
Duration of steroid use (days)	4.9 ± 2.2	5.1 ± 1.2	n.s.
Hospitalization (days)	7.7 ± 2.3	6.9 ± 1.9	n.s.
Total intermittent procaterol inhalations during hospitalization (number)	16.6 ± 5.4	10.5 ± 3.5	<0.001
ECP at discharge (mg/L)	33.7 ± 25.8 (n = 7)	30.7 ± 31.1 (n = 13)	n.s.

mPSL, methylprednisolone; BIS, budesonide inhalation suspension; ECP, eosinophil cationic protein; n.s., not significant.

**Figure 1:**
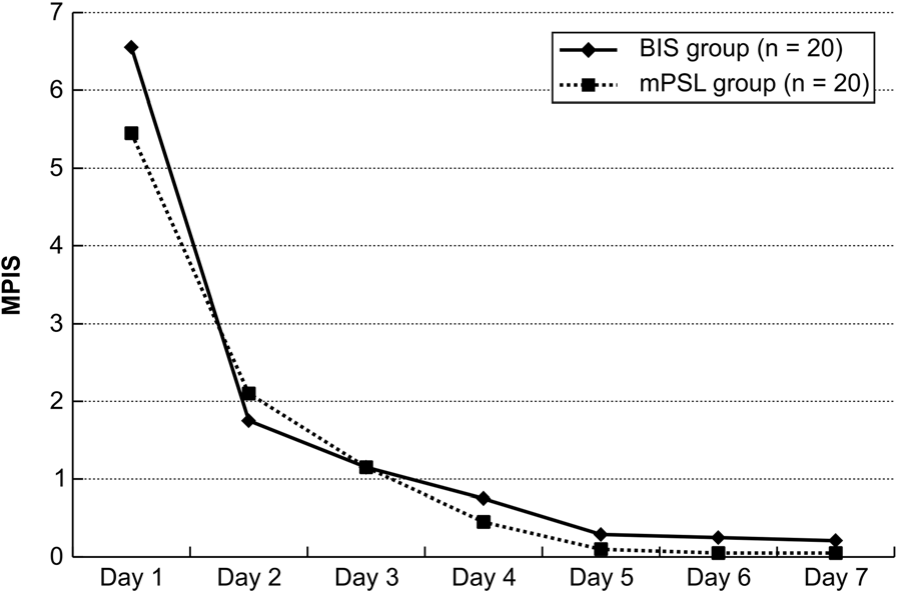
**Comparison of the Modified Pulmonary Index Score.** The Modified Pulmonary Index Score (MPIS) of the BIS group is shown by the closed line and that of the mPSL group is shown by the open line. No significant difference in the MPIS is seen between the BIS group and the mPSL group. Abbreviations: mPSL, methylprednisolone; BIS, budesonide inhalation suspension; MPIS, Modified Pulmonary Index Score.

The number of inhalations with procaterol during hospitalization was 10.5 ± 3.5 times in the BIS group, which was lower by approximately two-thirds than the value in the mPSL group (16.6 ± 5.4), and this difference was significant (*p* < 0.001). Cortisol levels at discharge were measured in 15 patients in the mPSL group and 18 patients in the BIS group. Cortisol levels could not be measured in five patients in the mPSL group and in two patients in the BIS group who were discharged on a weekend or national holiday. ECP levels at discharge could be measured only in seven patients in the mPSL group and 13 patients in the BIS group because of problems with sample processing. There was no significant difference in ECP levels at discharge (33.7 ± 25.8 μg/L in the mPSL group and 30.7 ± 31.1 μg/L in the BIS group). The respiratory status was evaluated using the MPIS (Figure 1), and no significant difference was observed between the groups at any time point.

**Figure 2:**
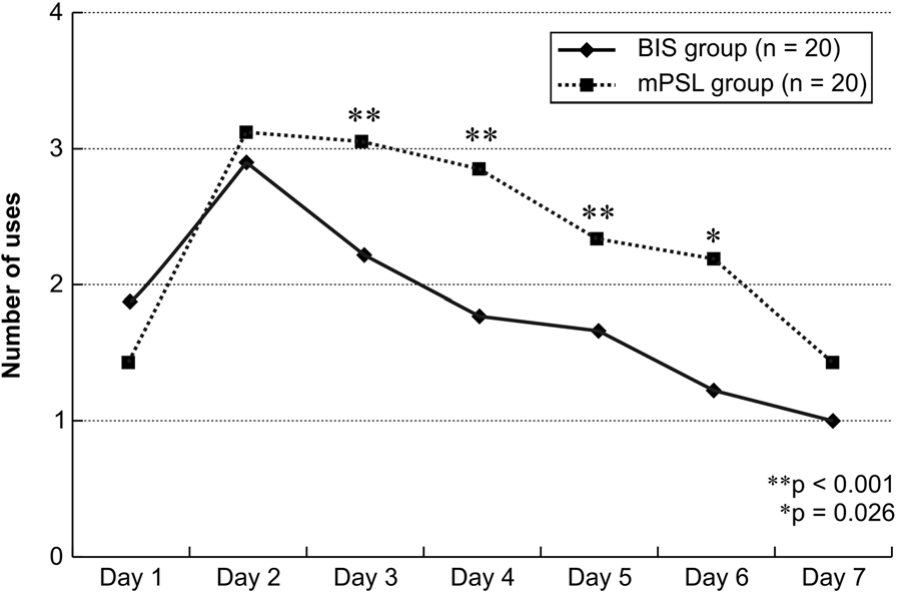
**Comparison of the frequency of using procaterol inhalation per day.** The BIS group is shown by the closed line, and the mPSL group is shown by the open line. The frequency of procaterol inhalation on days 3, 4, 5 and 6 is significantly less in the BIS group than in the mPSL group. Abbreviations: mPSL, methylprednisolone; BIS, budesonide inhalation suspension.

Figure 2 shows a comparison of the number of inhalations during hospitalization. Patients in the mPSL group required the use of an inhaled bronchodilator significantly more often on days 3 to 6 compared with the BIS group. The number of inhalations in the mPSL and BIS groups was 3.1 and 2.2 times on day 3 (p < 0.001), 2.8 and 1.8 times on day 4 (p < 0.001), 2.3 and 1.6 times on day 5 (p < 0.001), and 2.2 times and 1.2 times on day 6 (p = 0.026), respectively. No patient in either the mPSL or the BIS group required additional oxygen administration.

**Figure 3:**
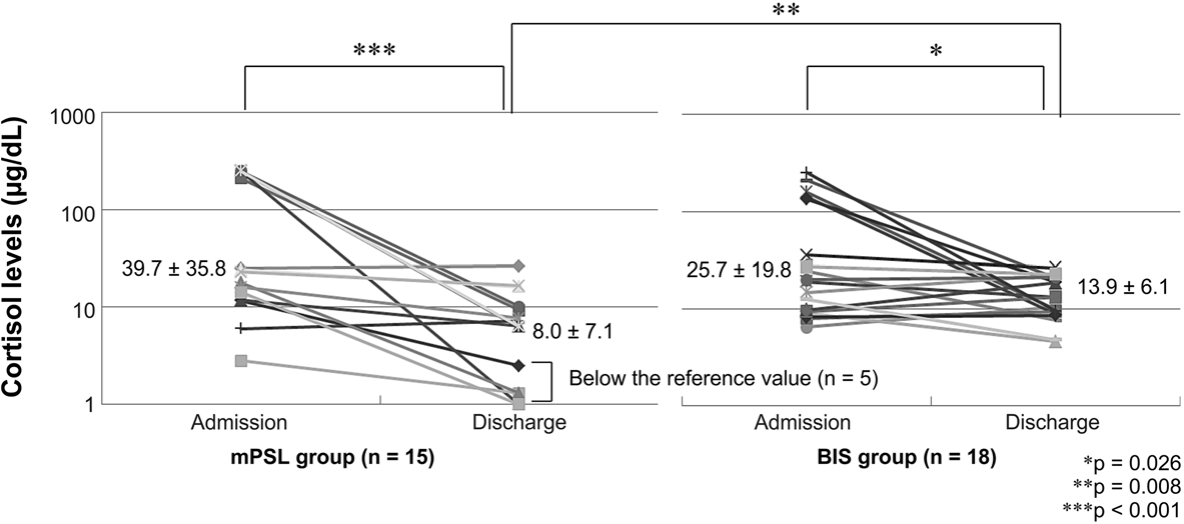
**Comparison of plasma cortisol levels.** The plasma cortisol level at discharge is significantly higher in the BIS group than in the mPSL group. Abbreviations: mPSL, methylprednisolone; BIS, budesonide inhalation suspension.

Figure 3 shows a comparison of cortisol levels before and after hospitalization. Cortisol levels at discharge decreased significantly compared with preadmission levels in both the mPSL and BIS groups. There was no significant difference between the two groups in terms of cortisol levels on admission, but at discharge, the levels were significantly higher in the BIS group (13.9 ± 6.8 μg/dL) than in the mPSL group (8.0 ± 7.1 μg/dL; *p* = 0.008).

Cortisol levels were below the reference value of 4 mg/dL in a significantly higher number of patients in the mPSL group (5 of 15) than in the BIS group (0 of 18; Fisher exact test, *p* = 0.013). Patients in whom cortisol levels were below the reference value were retested as outpatients after 1 month, and they all had normal cortisol levels (8.0–22.1 mg/dL).

## Discussion

We found that treatment with BIS is equivalent to or better than that with mPSL for moderate bronchial asthma attacks. In this study, patients treated with BIS required less frequent use of a bronchodilator, and the use of BIS did not cause suppression of adrenocortical function, which is often seen in steroid treatment.

The number of inhalations in the mPSL groups was higher than that in the BIS groups on days 3, 4, 5, and 6. We consider it unlikely that this was the result of milder wheezing episodes in the BIS group rather than an effect of the BIS itself. The MPIS of the BIS group was not lower than that of the mPSL group on day 1 and remained nearly constant from day 1 to day 7.

There have been a number of previous reports on the efficacy of BIS for acute exacerbations of bronchial asthma. Bacharier *et al.* reported that inhalation of 2 mg/day of BIS prevented aggravation of moderate-to-severe intermittent wheezing compared with a placebo [6]. A review by Schramm *et al.* also concluded that inhaled budesonide might have equivalent efficacy to oral steroids for acute attacks of bronchial asthma [7]. A meta-analysis by Xin-Ming *et al.* reported a similar rate of hospitalization in patients treated with inhaled budesonide and those treated with oral systemic corticosteroids [8]. It has also been reported that there was no significant difference between continuous daily administration of budesonide and intermittent administration of budesonide (1 mg) twice daily for one week at the time of an attack in terms of suppressing asthma attacks in preschool children with recurrent wheezing [9].

In a study on moderate-to-severe attacks, Matthews *et al.* compared 46 patients aged from 5 to 16 years with severe asthma attacks, not serious enough to be life-threatening, who were treated with 2 mg/kg of prednisolone every 24 hours and those treated with 2 mg/kg of nebulized budesonide every 8 hours [10]. There was a significantly greater improvement in forced expiratory volume per second in the nebulized-budesonide group 24 hours after admission. The authors stated that nebulized budesonide was at least as effective as oral steroids. Devidayal *et al.* studied 80 children aged from 2 to 12 years with moderate-to-severe attacks, and they compared patients who underwent three inhalations with 800 μg of BIS and 0.15 mg/kg of salbutamol at 30-minute intervals with those treated with 2 mg/kg of oral prednisolone and three inhalations with 0.15 mg/kg of salbutamol at 30-minute intervals. They found that SpO_2_, number of breaths, pulmonary function, and dyspnea score improved significantly in the BIS group after 2 hours, and that those patients could be discharged earlier [11]. Milani e*t al.* performed a double-blind study of three groups of children aged from 2 to 7 years with severe bronchial asthma attacks only, treated with 2 mg of BIS, 1 mg/kg of oral prednisolone, and placebo, and they found that although BIS and prednisolone resulted in an equivalent amelioration of symptoms, SpO_2_ improved more rapidly with oral prednisolone [12]. They stated that inhalation treatment may be insufficient in cases of severe bronchial asthma attacks, and that oral steroids may be more effective as their absorption can be assured. Conversely, in moderate attacks in which adequate inhalation is feasible, BIS may be of equivalent therapeutic efficacy to oral steroids. This may support our finding that BIS was useful for moderate attacks. However, it may not be effective for severe attacks, and further study is required. In the present study, we used a lower dose of BIS (0.5 mg/treatment) than the doses of 0.8 to 2 mg reported in other studies. However, we observed no significant difference between the therapeutic effect of BIS and that of systemic administration of mPSL. As noted earlier, nurses ensured that caregivers were able to properly administer BIS. This meant that there was little variation in the inhalation procedure, which may have ensured the full therapeutic effect of BIS.

It is important to consider the effect of steroid administration on adrenocortical function. Shapiro *et al.* administered inhalation treatment with the doses of 0.25 mg, 0.5 mg, and 1.0 mg of budesonide twice a day for 12 weeks to 178 children aged from 4 to 8 years with inhaled steroid-dependent bronchial asthma, and they found no significant difference between the three experimental groups and the placebo group in terms of cortisol levels and adrenocorticotropic hormone challenge test results [13]. The maximum daily dose of BIS of 1.5 mg used in the present study was lower than the maximum dose of 2 mg used in the other study, and the duration of administration of less than 1 week in the present study was shorter than the 12 weeks in the other study. The dose of BIS used in the present study should therefore not have a major effect on adrenocortical function.

In the present study, although cortisol levels were not below the reference value in any of the patients in the BIS group, they were below the reference range in one-third of the patients in the mPSL group. Hedlin *et al.* compared cortisol levels in children aged from 1 to 3 years treated for 10 days with oral betamethasone or high-dose inhaled budesonide (800–1600 μg/day). They found that cortisol levels decreased in patients who received betamethasone, but not in those treated with budesonide [14].

Treatment of moderate bronchial asthma attacks with BIS does not cause adrenocortical suppression, which is a common side effect in systemic administration of mPSL. Moreover, treatment of moderate bronchial asthma attacks with BIS is equally effective as standard systemic administration of intravenous steroids in the hospital. Because BIS can be administered at home if a nebulizer is available, it may allow home treatment of asthma without the common side effects of steroid therapy. Thus, this treatment may also help reduce the medical costs associated with hospitalization for bronchial asthma attacks. However, it may not be effective if appropriate inhalation procedures are not followed. Furthermore, whereas BIS appears to be effective for moderate attacks, evidence is lacking with respect to severe cases. In summary, BIS may be considered as a treatment option for acute attacks in moderate cases providing that appropriate inhalation procedures are observed.

Nonetheless, there are several limitations to this study. This was not a double-blind study and a double-blind study is required. Approximately half of the patients in both groups had already been treated with intravenous hydrocortisone sodium succinate prior to admission, but none of them had been treated more than once and the maximum dose was 7 mg/kg; therefore, the effect was limited because the patients were all administered hydrocortisone sodium succinate, which has a half-life of only 90 minutes. There were some limitations regarding the cortisol assessments, because this study ended 2 days after the last dose and the effect may have thus been underestimated. It should also be noted that the sample size was small and thus further study is needed. In addition, the results may not be generalized to children of any age and of those with severe asthma attacks.

## Conclusions

We compared systemic administration of mPSL and treatment with BIS in patients hospitalized for moderate bronchial asthma attacks and showed that the efficacy of BIS was equivalent to or better than that of mPSL. In addition, BIS enabled less frequent usage of inhaled bronchodilators and did not cause side effects such as adrenal suppression. Although future studies to confirm its efficacy for severe bronchial asthma attacks are required, it may be a valid therapeutic option for moderate bronchial asthma attacks as long as appropriate inhalation procedures are observed.

### Ethical considerations

After collecting data on all patients, the study was registered as a clinical trial (UMIN000012722). Written informed consent was obtained from the legal guardians of all patients, and the study protocol was approved by the Ethics Committee of Sagamihara National Hospital.

## Abbreviations

BIS: Budesonide inhalation suspension
mPSL: Methylprednisolone
DSCG: Disodium cromoglycate
JPGL2008: Japanese Pediatric Guideline for the Treatment andManagement of Asthma 2008
MPIS: Modified Pulmonary Index Score
CRP: C-reactive protein
ECP: Eosinophil cationic protein
SpO_2_: Percutaneous oxygen saturation
ACTH: Adrenocorticotropic hormone
